# Factors associated with the development of septic shock in patients with candidemia: a post hoc analysis from two prospective cohorts

**DOI:** 10.1186/s13054-020-2793-y

**Published:** 2020-03-26

**Authors:** Matteo Bassetti, Antonio Vena, Marco Meroi, Celia Cardozo, Guillermo Cuervo, Daniele Roberto Giacobbe, Miguel Salavert, Paloma Merino, Francesca Gioia, Mario Fernández-Ruiz, Luis Eduardo López-Cortés, Benito Almirante, Laura Escolà-Vergé, Miguel Montejo, Manuela Aguilar-Guisado, Pedro Puerta-Alcalde, Mariona Tasias, Alba Ruiz-Gaitán, Fernando González, Mireia Puig-Asensio, Francesc Marco, Javier Pemán, Jesus Fortún, Jose Maria Aguado, Alejandro Soriano, Jordi Carratalá, Carolina Garcia-Vidal, Maricela Valerio, Assunta Sartor, Emilio Bouza, Patricia Muñoz

**Affiliations:** 1 Infectious Diseases Clinic, Department of Medicine, University of Udine and Azienda Sanitaria Universitaria Integrata, Piazzale Santa Maria della Misericordia 15, 33010 Udine, Italy; 2 Department of Health Sciences, University of Genoa, Genoa, Italy; 3 Clinica Malattie Infettive, Ospedale Policlinico San Martino-IRCCS, Genoa, Italy; 4 Hospital Clínic, IDIBAPS (Institut d’Investigacions biomèdiques Agust Pi i Sunyer), Universitat de Barcelona, Barcelona, Spain; 5 Hospital Universitari de Bellvitge, IDIBELL (Institut D’Investigació Biomèdica de Bellvitge), Universitat de Barcelona, Barcelona, Spain; 6 Hospital Universitari I Politecnic “La Fe”, Valencia, Spain; 7 Hospital Universitario Clínico “San Carlos”, Madrid, Spain; 8 Hospital Universitario “Ramón y Cajal”, Madrid, Spain; 9 Hospital Universitario “12 de Octubre”, Instituto de Investigación Hospital “12 de Octubre” (i+12), Universidad Complutense de Madrid, Madrid, Spain; 10 Unidad Clínica de Enfermedades Infecciosas y Microbiología Clínica, Hospital Universitario Virgen Macarena/Instituto de Biomedicina de Sevilla (IBiS)/Universidad de Sevilla/Centro Superior de Investigaciones Científicas, Seville, Spain; 11 Hospital Universitari Vall d’Hebron, VHIR (Vall d’Hebron Institut de Recerca), Universitat Autònoma de Barcelona, Barcelona, Spain; 12 Hospital Universitario “Cruces”, Bilbao, Spain; 13 Hospital Universitario “Virgen del Rocío”, Sevilla, Spain; 14 Clinical Microbiology and Infectious Diseases, Hospital General Universitario Gregorio Marañón, Madrid, Spain; 15 Instituto de Investigación Sanitaria, Hospital Gregorio Marañón, Madrid, Spain; 16 Microbiology Unit, Azienda Sanitaria Universitaria Integrata Santa Maria della Misericordia, Udine, Italy; 17 Medicine Department School of Medicine, Universidad Complutense de Madrid, Madrid, Spain; 18 CIBER Enfermedades Respiratorias-CIBERES (CB06/06/0058), Udine, Spain

**Keywords:** Candidemia, Septic shock, Intra-abdominal candidiasis, Risk factors

## Abstract

**Background:**

Almost one third of the patients with candidemia develop septic shock. The understanding why some patients do and others do not develop septic shock is very limited. The objective of this study was to identify variables associated with septic shock development in a large population of patients with candidemia.

**Methods:**

A post hoc analysis was performed on two prospective, multicenter cohort of patients with candidemia from 12 hospitals in Spain and Italy. All episodes occurring from September 2016 to February 2018 were analyzed to assess variables associated with septic shock development defined according to The Third International Consensus Definition for Sepsis and Septic Shock (Sepsis-3).

**Results:**

Of 317 candidemic patients, 99 (31.2%) presented septic shock attributable to candidemia. Multivariate logistic regression analysis identifies the following factors associated with septic shock development: age > 50 years (OR 2.57, 95% CI 1.03–6.41, *p* = 0.04), abdominal source of the infection (OR 2.18, 95% CI 1.04–4.55, *p* = 0.04), and admission to a general ward at the time of candidemia onset (OR 0.21, 95% CI, 0.12–0.44, *p* = 0.001). Septic shock development was independently associated with a greater risk of 30-day mortality (OR 2.14, 95% CI 1.08–4.24, *p* = 0.02).

**Conclusions:**

Age and abdominal source of the infection are the most important factors significantly associated with the development of septic shock in patients with candidemia. Our findings suggest that host factors and source of the infection may be more important for development of septic shock than intrinsic virulence factors of organisms.

## Introduction

*Candida* species has been reported as the most common cause of fungal disease in septic shock, affecting 8–10% of these patients [1–3]. Moreover, among patients developing septic shock, candidemia is actually considered the clinical condition with the highest attributable mortality, ranging from 54 to 66% [4–6]. The survival of these patients is strictly related to a timely control of the source and appropriate antifungal treatment [4, 5].

While candidemia has long been recognized as a trigger for septic shock development, our understanding of why patients with candidemia develop or not septic shock has not been well addressed [7]. Literature on the topic is very limited and difficult to interpret due to the low number of patients included and the inconclusive results reported [7].

Therefore, the aim of our study was to assess factors associated with the development of septic shock in the context of two large multicenter studies of patients with candidemia.

## Materials and methods

### Study design, setting, and population

In the present report, we included data coming from two separate studies. First is a prospective cohort assembled within a quasi-experimental study aimed at assessing the impact of an evidence-based intervention bundle to improve the clinical management of candidemia (the CANDI-Bundle Study) that was conducted from September 2016 to February 2018 in 11 hospitals from Spain. Second is a prospective observational study on candidemia that was conducted during the same period at the University Hospital Santa Maria della Misericordia in Udine, Italy. The full results from both cohorts are currently undergoing detailed analysis.

In order to ensure comparability, the same inclusion and exclusion criteria, study definitions, patient follow-up, and data collection procedures were applied in both studies. However, for this post hoc analysis, we excluded patients aged < 18 years old and those who had mixed infection (concomitant blood culture positive for a bacterial pathogen).

The study was approved by the local institutional review boards, and written patient consent was not required because of observational nature of this study.

### Clinical data and definitions

Data were collected from patients’ hospital charts and the laboratory database, which contains complete profiles for all candidemic patients. The main outcome measured was development of septic shock attributable to candidemia. Septic shock patients and non-septic shock subgroups were compared to identify variables associated with septic shock development.

The following variables were explored as possibly associated with septic shock development:

i. For patient variables, we considered age, sex, hospital ward stay at the time of infection, Charlson comorbidity index [8]),underlying diseases, immunosuppressive therapy, history of previous abdominal surgery (30 days preceding candidemia onset), presence of central venous catheter (CVC) at the time of candidemia, total parenteral nutrition, and prior exposure to antimicrobial or antifungal therapy (within previous 30 days).
ii. For infection variables, we considered source of infection, pathogen species, and antifungal resistance.

All patients were followed until discharge or death and were assessed for survival on day 30.

### Definitions

An episode of candidemia was defined as at least one peripheral blood culture positive for *Candida* spp. Candidemia onset was defined as the date of collection of the first blood culture yielding the study isolate.

Septic shock was determined according to The Third International Consensus Definition for Sepsis and Septic Shock (Sepsis-3) [9]. All clinical and laboratory data defining septic shock were assessed within 24 h of the first positive blood culture yielding *Candida* spp. Patients were classified as having septic shock attributable to *Candida* infection if vasopressors were required to maintain a mean arterial pressure of ≥ 65 mmHg and serum lactate level greater than 2 mmol/L [9]. Patients who required vasopressors ≥ 24 h prior to the collection of a blood culture subsequently positive for *Candida* species were included in the non-septic shock group.

As for the source of infection, CVC-related candidemia was defined according to current guidelines [10, 11]. The urinary tract was considered to be the portal of entry in patients with urological predisposing conditions (i.e., manipulation or obstruction of the urinary tract) and evidence of urinary tract infection caused by the same *Candida* spp. [12]. The abdomen was considered to be the origin of the candidemia when a patient had evidence of abdominal infection and (i) a positive culture from the intra-abdominal space was obtained during surgery or by needle aspiration or (ii) no other apparent sources of candidemia were detected [13–15]. When a source of candidemia could not be identified, candidemia was defined as “primary.”

Initial antifungal therapy was considered as adequate if at least one active drug according to in vitro susceptibility results was initiated at an appropriate dosage [10] within the first 24 h after blood culture was obtained.

Source control included CVC withdrawal and invasive procedures to resolve urinary tract obstruction and intra-abdominal abscess drainage, depending on the source of candidemia. Source control was considered adequate when performed within 24 h from candidemia onset.

Neutropenia was defined as an absolute neutrophil count< 500 cells/mm^3^ at the onset of candidemia.

### Microbiological studies

*Candida* specie identification and in vitro antifungal activity were assessed at participating hospitals using local routine methods. Currently, two sets of two blood samples were collected from patients with a suspected bloodstream infection. The blood samples were processed using a BACTEC 9240 system (Becton– Dickinson Microbiology Systems, Franklin Lakes, NJ, USA) or BACTE 860 system or BacTAlert (BioMérieux SA, Marcy L’Etoile, France) with an incubation period of 5 days. If yeast cells were observed after microscopic examination of a Gram stain, blood bottles were subcultured into Sabouraud agar plates (BD BBL StrackerTM PlatesTM, Heidelberg, Germany) and chromogenic media (ChromAgar BioMerieux SA, Paris, France). In vitro antifungal activity was studied by a commercial microdilution method (YeastOne®Sensitre®, TREK Diagnostic Systems Ltd., East Grinstead, UK) or E test (BioMérieux SA, Paris, France), in accordance with the manufacturer’s instructions.

### Statistical analysis

Categorical variables are expressed as absolute numbers and relative frequencies. Quantitative variables are presented as means and standard deviation (SD) if normally distributed or as median and interquartile range (IQR) if non-normally distributed. We compared continuous variables between patients with or without septic shock using the Student *t* test for normally distributed variables and the Mann-Whitney *U* test for non-normally distributed variables. Categorical variables were evaluated by using the chi-square or the two-tailed Fisher exact test.

Variables associated with septic shock due to candidemia in univariate analysis (*p* ≤ 0.20) were included in a logistic regression analysis, and a backward stepwise approach was used to identify those independently associated with septic shock development (*p* < 0.05). All statistical analyses were performed by using SPSS Statistics for MAC, Version 22.0 (SPSS Inc., Chicago, IL, USA).

## Results

A total of 317 episodes of candidemia were included in the study. Overall, 99 of the 317 patients (31.2%) fulfilled the criteria for septic shock within the first 24 h after blood culture was drawn, and 34.3% of them died in 30 days.

### Clinical characteristics of study population

Demographics and baseline clinical features of study population are shown in Table 1. Mean age was 65.4 years, and 184 patients were men (58.0%). Overall, 63 out of 317 (19.9%) episodes of candidemia occurred in intensive care unit (ICU), and 254 (80.1%) occurred in patients hospitalized in general wards, including 150 (47.3%) in internal medicine and 104 (32.8%) in surgical wards.

**Table 1:** Comparison of the main demographic and clinical characteristics of study population

Variable	All episodes, *N* = 317 (%)	Non-septic shock, *N* = 218 (%)	Septic shock, *N* = 99 (%)	*p* value
Demographics				
Sex, male	184 (58.0)	141 (64.7)	43 (43.4)	0.17
Mean age (± SD), years	65.4 ± 17.0	65.2 ± 18.6	66.9 ± 15.2	0.77
Age ≥ 50 years	275 (86.4)	183 (83.9)	92 (92.9)	**0.03**
Median hospital stay until *Candida* BSI (IQR), days	16 (7–31)	17 (6–32)	14 (8–28)	0.18
Hospital ward stay at the time of candidemia onset				
General ward	254 (80.1)	192 (88.1)	62 (62.6)	**0.001**
Intensive care unit	63 (19.9)	26 (11.9)	37 (37.4)	
Charlson comorbidity index	3.4 ± 2.7	3.4 ± 2.6	3.3 ± 2.8	0.71
Underlying condition				
Solid tumor	114 (35.9)	77 (35.3)	37 (37.4)	0.80
Cardiovascular disease	102 (32.2)	69 (31.7)	33 (33.3)	0.79
Diabetes mellitus	90 (28.4)	57 (26.1)	33 (33.3)	0.22
Chronic Lung disease	51 (16.1)	35 (16.1)	16 (16.2)	1
Chronic kidney failure	50 (15.8)	32 (14.7)	18 (18.2)	0.50
Chronic liver disease	41 (12.9)	27 (12.4)	14 (14.1)	0.74
Solid organ transplantation	27 (8.5)	19 (8.7)	8 (8.1)	1
Hematological malignancy	20 (6.3)	16 (7.3)	4 (4.0)	0.32
Hematopoietic stem cell transplantation	9 (2.8)	8 (3.7)	1 (1.0)	0.28
Risk factors for candidemia				
Previous antibiotic therapy	297 (93.6)	202 (92.7)	95 (96.0)	0.32
Central venous catheter	225 (71.0)	150 (68.8)	75 (75.8)	0.23
TPN during candidemia	158 (49.8)	101 (46.3)	57 (57.6)	0.07
Previous corticosteroid therapy	72 (22.7)	51 (23.4)	21 (22.1)	0.77
Abdominal surgery	85 (26.8)	52 (23.9)	33 (33.3)	0.10
Immunosuppressive therapy	47 (14.8)	36 (16.5)	11 (11.1)	0.23
Neutropenia	10 (3.2)	9 (4.1)	1 (1.0)	0.18
Previous antifungal treatment	49 (15.4)	37 (17.0)	12 (12.1)	0.32
Source of infection				
Central venous catheter	141 (44.5)	101 (46.3)	40 (40.4)	0.33
Primary	98 (30.9)	73 (33.5)	25 (25.3)	0.15
Abdomen	39 (12.3)	19 (8.7)	20 (20.2)	**0.006**
Urinary tract	23 (7.3)	18 (8.3)	5 (5.1)	0.36
Infective endocarditis	3 (0.9)	1 (0.5)	2 (2.0)	0.23
Other	13 (4.1)	6 (2.8)	7 (7.1)	0.12
*Candida* species				
*C*. *albicans*	134 (42.3)	91 (41.7)	43 (43.4)	0.80
*C*. *glabrata*	58 (18.3)	38 (17.4)	20 (20.2)	0.63
*C*. *parapsilosis*	55 (17.4)	40 (18.3)	15 (15.2)	0.52
*C. tropicalis*	29 (9.1)	22 (10.1)	7 (7.1)	0.52
*C. krusei*	6 (1.9)	4 (1.8)	2 (2.0)	1
*C. lusitaniae*	4 (1.3)	2 (0.9)	2 (2.0)	0.59
*C. auris*	27 (8.5)	18 (8.3)	9 (9.1)	0.86
Other	13 (4.1)	11 (5.0)	2 (2.0)	0.35
Resistant strains				
Fluconazole	45 (14.1)	29 (13.3)	16 (16.2)	0.73
Echinocandins	3 (0.9)	2 (0.9)	1 (1.0)	1

*BSI* bloodstream infection, *IQR* interquartile range, *SD* standard deviation, *TPN* total parenteral nutrition

The most common underlying condition was solid tumor (35.9%) followed by cardiovascular disease (32.2%) and diabetes mellitus (28.4%). Almost all patients had recently received antibiotic therapy (within the previous 1 month). A CVC was in place in 225 out of 317 patients (71.0%), with 158 of them receiving total parenteral nutrition at the time of candidemia onset. The most prevalent source of infection was the CVC (44.5%) followed by the abdomen that was detected in 39 patients (12.3%). Source of candidemia remained unknown in 98 (30.9%) patients.

As for species, *C*. *albicans* was the most frequent one (42.3%) followed by *C. glabrata* (18.3%), *C. parapsilosis* (17.4%), and *C. tropicalis* (9.1%).

### Variables associated with septic shock attributable to candidemia

Patients with septic shock attributable to candidemia (99 patients) and without septic shock (218 patients) were compared (Table 1). There were no significant differences regarding sex, Charlson comorbidity index, underlying disease, and risk factors for candidemia and *Candida* species. However, at univariate analysis, factors associated with development of septic shock were as follows: age older than 50 years (92.9% vs 83.9%; *p* = 0.03), hospitalization in ICU at the time of candidemia (37.4% vs 11.9%, *p* = 0.001), and an abdominal source of the infection (20.2% vs 8.7%; *p* = 0.006). A multivariate analysis (Table 2) showed that older age (OR 2.57, 95% CI 1.03–6.41, *p* = 0.04) and an abdominal source of the infection (OR 2.18, 95% CI 1.04–4.55, *p* = 0.04) were independently associated with the development of septic shock in candidemic patients. By contrast, being admitted to a general ward at the time of candidemia onset was considered as a protective factor (OR 0.21, 95% CI 0.12–0.44, *p* = 0.001).

**Table 2:** Risk factors for development of septic shock at candidemia onset. Multivariate analysis

Variable	Odds ratio	95% confidence interval	*p* value
Age > 50 years	2.57	1.03–6.41	**0.04**
Intra-abdominal source of the infection	2.18	1.04–4.55	**0.04**
Male sex	1.33	0.78–2.26	0.29
Charlson comorbidity index	0.99	0.91–1.10	0.92
General ward stay at the time of candidemia onset	0.21	0.12–0.44	**0.001**

Comparison between patients with and without intra-abdominal origin of the infection is reported in Supplementary material 1. As for infection management, echinocandin was the most frequent initial antifungal agent, prescribed in 57.7% of cases, followed by azoles in 35.3% of patients. Although rates of initial echinocandin therapy (65.3% vs 60.9%) or initial azole therapy (32.7% vs 39.1%) were comparable, patients with an intra-abdominal origin received an adequate initial antifungal treatment more often (38.1 vs 61.5, *p* = 0.008). No differences regarding the rates of adequate source control (43.4% vs 40.5%, *p* = 0.86) were observed between groups.

### Prognostic factors for 30-day mortality

**Fig. 1:**
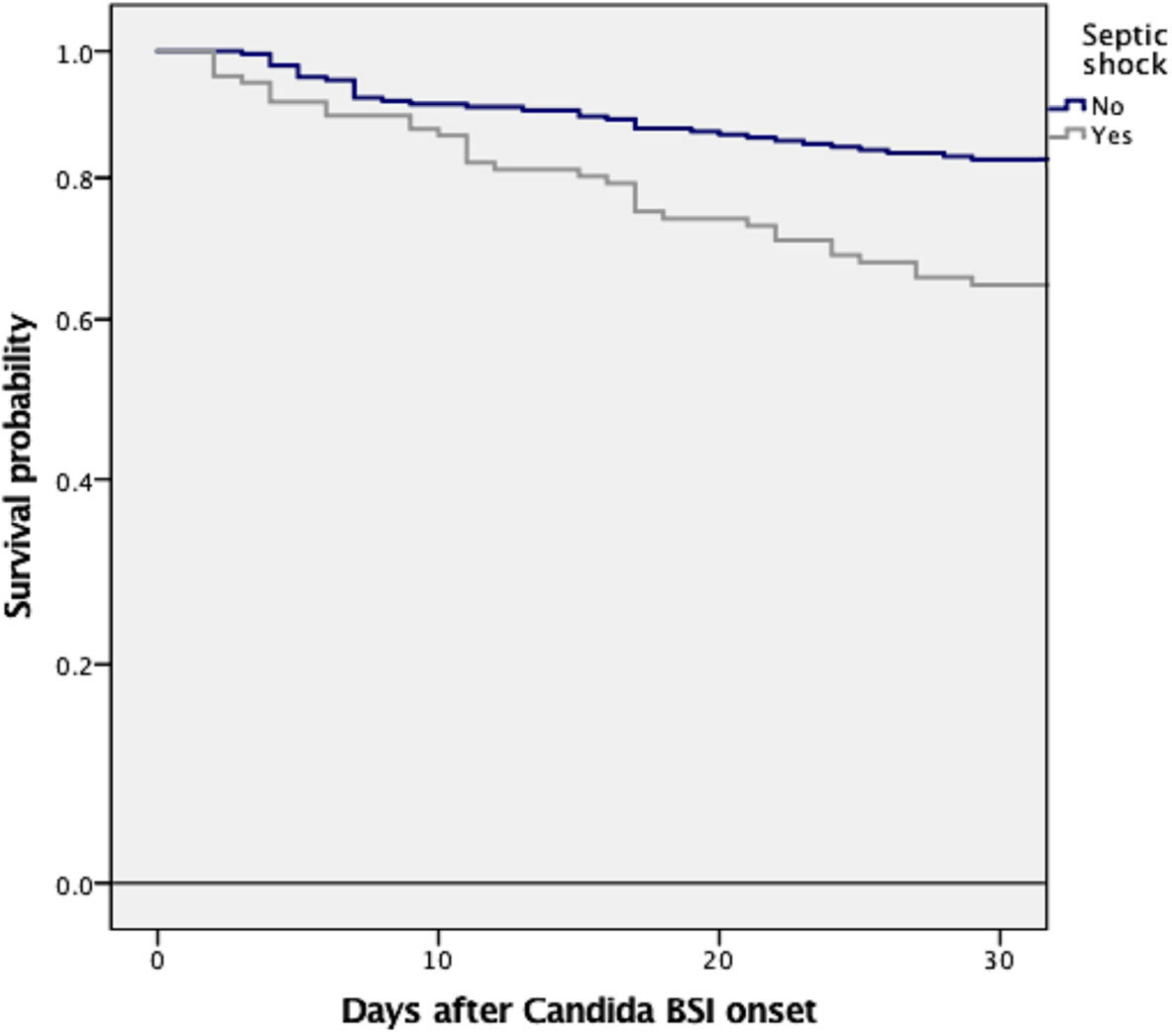
Thirty-day mortality of candidemic patients according to the development or not of septic shock, log rank ≤  0.001

We compared candidemic patients who died at 30 days (70 patients) with those who survived (247 patients). Septic shock attributable to candidemia was present in 48.6% of patients who died (34/70) in comparison to 26.3% (65/247 patients) who survived (*p* = 0.001) (Table 3). The Kaplan-Meier curves in Fig. 1 demonstrate that patients with septic shock attributable to candidemia had a significantly greater likelihood of dying compared to patients not developing septic shock at candidemia onset.

**Table 3:** Risk factors for 30-day mortality (univariate and multivariate analysis)

Variable	All episodes, *N* = 317 (%)	Alive, *N* = 247 (%)	Died, *N* = 70 (%)	*p*	OR	95% CI	*p*
Demographics							
Sex, male	184 (58.0)	154 (62.3)	46 (61.4)	0.89	–	–	–
Age ≥ 50 years	275 (86.8)	212 (85.8)	63 (90.0)	0.43	–	–	–
Mean age (± SD), years	65.4 ± 17.0	64.8 ± 16.8	67.2 ± 17.7	0.30	–	–	–
Median hospital stay until *Candida* BSI (IQR), days	16 (7–31)	15 (6–29)	20 (10–34)	0.09	–	–	
Hospital ward							
General ward	254 (80.1)	201 (84.1)	47 (67.1)	**0.003**	1.9	0.93–4.30	0.78
Intensive care unit	63 (19.9)	38 (15.9)	23 (32.9)				
Charlson comorbidity index	3.4 ± 2.7	3.0 ± 2.4	4.6 ± 3.1	**< 0.001**	**1.28**	**1.13–1.45**	**< 0.001**
Underlying condition							
Solid tumor	114 (35.9)	91 (36.8)	23 (32.9)	0.57	–	–	–
Cardiovascular disease	102 (32.2)	72 (29.1)	30 (42.9)	**0.04**	–	–	–
Diabetes mellitus	90 (28.4)	68 (27.5)	22 (31.4)	0.55	–	–	–
Chronic lung disease	51 (16.1)	36 (14.6)	15 (21.4)	0.20	–	–	–
Chronic kidney failure	50 (15.8)	36 (14.6)	14 (20.0)	0.27	–	–	–
Chronic liver disease	41 (12.9)	26 (10.5)	15 (21.4)	**0.02**	–	–	–
Solid organ transplantation	27 (8.5)	22 (8.9)	5 (7.1)	0.81	–	–	–
Hematological malignancy	20 (6.3)	15 (6.1)	5 (7.1)	0.78	–	–	–
Hematopoietic stem cell transplantation	9 (2.8)	6 (2.4)	3 (4.3)	0.41	–	–	–
Risk factors for candidemia							
Previous antibiotic therapy	297 (93.6)	232 (93.9)	65 (92.9)	0.78	–	–	–
Central venous catheter	225 (71.0)	171 (69.2)	54 (77.1)	0.23	–	–	–
TPN during candidemia	158 (49.8)	118 (47.8)	40 (57.1)	0.17	–	–	–
Previous corticosteroid therapy	72 (22.7)	53 (21.5)	19 (27.1)	0.33	–	–	–
Abdominal surgery	85 (26.8)	65 (26.3)	20 (28.6)	0.76	–	–	–
Immunosuppressive therapy	47 (14.8)	39 (15.8)	8 (11.4)	0.45	–	–	–
Neutropenia	10 (3.2)	7 (2.8)	3 (4.3)	0.46	–	–	–
Previous antifungal treatment	49 (15.4)	37 (15.0)	12 (17.1)	0.71	–	–	–
Source of infection							
Central venous catheter	141 (44.5)	122 (49.9)	19 (27.1)	**0.001**	0.42	0.20–0.88	0.02
Primary	98 (30.9)	71 (28.7)	27 (38.6)	0.14	–	–	
Abdomen	39 (12.3)	22 (8.9)	17 (24.3)	**0.001**	1.89	0.76–4.66	0.17
Urinary tract	23 (7.3)	22 (8.9)	1 (1.4)	**0.03**	0.11	0.12–1.02	0.05-
Infective endocarditis	3 (0.9)	3 (1.2)	0 (0)	1	–	–	–
Other	13 (4.1)	7 (2.8)	6 (8.6)	0.05	–	–	–
Septic shock	99 (31.2)	65 (26.3)	34 (48.6)	**0.001**	**2.14**	**1.08–4.24**	**0.02**
Initial adequate antifungal therapy	130 (41.0)	95 (38.5)	35 (50.0)	0.09	1.41	0.71–2.81	0.32
Adequate source control of the infection*	124/288 (43.1)	94/225 (41.8)	30/63 (47.6)	0.47	1.68	0.84–3.35	0.14
Hemodialysis after candidemia onset	7 (2.2)	2 (0.8)	5 (7.1)	**0.007**	**8.55**	**1.35–53.95**	**0.02**

*The source of the infection was susceptible of control in 288 patients

*BSI* bloodstream infection, *IQR* interquartile range, *SD* standard deviation, *TPN* total parenteral nutrition

Variables examined as risk factors for 30-day mortality are presented in Table 3. Multivariate logistic regression analysis demonstrated that a higher Charlson comorbidity index and septic shock attributable to candidemia and requiring hemodialysis after candidemia onset were independently associated with greater 30-day mortality, while CVC-related candidemia was independently associated with a lower risk of 30-day mortality (Table 3).

## Discussion

To the best of our knowledge, the current multicenter study is the largest one to identify variables associated with development of septic shock among patients with *Candida* bloodstream infection. We found a significant rate of septic shock among patients with candidemia (30%), and we also demonstrated that age more than 50 years old and an intra-abdominal origin of the infection are the most important independent factors associated with septic shock development.

The incidence of septic shock is only collaterally mentioned in reports on candidemia in the general populations and it ranges from 12.9 to 49.0% in recent studies [16–18]. However, only three series have specifically focused on the incidence of septic shock among candidemic patients. Patel et al. [19] in a single center retrospective study that ended in 2007 documented septic shock in 23% of patients with candidemia. The prevalence was higher in two cohorts of critically ill patients with candidemia in which septic shock developed in ~ 35% of them [7, 20]. Ours is the first study to analyze the prevalence of septic shock attributable to candidemia, in the light of the new Sepsis-3 definition [9]. With these new criteria, we found that septic shock occurred in almost one third of unselected candidemic patients.

We also found a significant difference in term of prevalence between ICU patients and those admitted to internal medicine and surgical ward, with a nearly two times greater proportion of septic shock in ICU patients. Although such difference was not unexpected, an underestimation of the number of cases of septic shock outside ICU should not be excluded. Regarding this aspect, new Sepsis-3 definitions showed low sensitivity in non-critically ill patients and performed poorly as a screening tool for early identification of sepsis outside ICU [21–23] Moreover, many patients admitted to internal medicine and surgical ward are frequently affected by multiple pre-existing underlying diseases [24–28]. In such circumstance, worsening of clinical conditions can be often misled to the progression of the underlying disease rather than to infection, as suggested by the low number of patients who were admitted to ICU after candidemia onset in our study (~ 7%).

Factors predisposing candidemic patients to develop septic shock have been reported in just one retrospective study including only 15 patients with septic shock [7]. Reflecting this weakness, this study identified no conventional factors associated with septic shock development other than the time spent in ICU before candidemia onset.

Our data suggest that, apart from older age, the only variable associated with septic shock in candidemic patient was an intra-abdominal origin of the infection. This result supports recent experimental observations that intestinal abundance of *Candida* may be associated with an increased sepsis severity, perhaps through cytokine storm induction and/or decreased macrophage killing activity [29–31]. The fact that patients with other source of the infection (i.e., CVC or urinary tract) received a similar rate of adequate source control of candidemia (43.4% vs 40.5%, *p* = 0.86) and even a lower rate of adequate antifungal treatment (38.1% vs 61.5%, *p* = 0.008) is further consistent with this intriguing explanation. Although we do not have clear answer regarding to the best approach for reducing the incidence of septic shock in candidemic patients, we believe that new diagnostic strategies investigating the role of serological biomarkers such as β-d-glucan or T2MR [32–35] should be applied in order to early identify patients at risk of intra-abdominal candidiasis. Future studies addressing risk factors for developing intra-abdominal candidiasis are also needed to better clarify the best empiric or pre-emptive therapeutic approach.

With regard to *Candida* species as predisposing factor, Guzman et al. did [36] not find any significant differences [7]. Despite our study including 27 episodes of *C*. *auris* candidemia (8.5%) that is typically associated with a high degree of virulence [37], we confirm previous results, thus suggesting that host factors may be more important than intrinsic factors of organisms. Antifungal resistance was not an issue predisposing to septic shock in our study, since only 14.1% of the patients had fluconazole resistant strains and half of them received adequate definitive antifungal treatment.

Of interest, in the present report, the overall 30-day mortality rate in patients with septic shock attributable to candidemia was 34.3%, which is significantly lower than that observed in two previous studies performed in Europe and St. Louis, where mortality rate remained around 50–60% [4, 5]. The difference in septic shock definitions [9], improvements in fungal diagnosis [33, 34], and the widespread implementation of sepsis bundle in recent years [38–40] may explain the lower incidence of mortality observed in this study.

As for risk factors for mortality, our findings are consistent with those recently reported by other investigators [41] who found increased severity of the underlying conditions (higher Charlson comorbidity index), clinical presentation of candidemia (septic shock), and signs of organ dysfunction (hemodialysis after candidemia onset) as risk factors for late mortality (30 days after candidemia onset).

Although ours is the largest study published to date regarding variables associated with septic shock in candidemic patients, it has some limitations that should be addressed. We only examine patients who develop septic shock within the first 24 h from the first positive blood culture for *Candida*. Therefore, we cannot rule out that some patients presented with a septic shock in a later stage of the infection. Furthermore, we did not include, in the risk factors analysis for mortality, other core elements of general supportive care that are crucial for an adequate septic shock management (need for mechanical ventilation, use of intravenous fluids and oxygen therapy). Therefore, additional studies specifically designed to investigate risk factors predicting septic shock development in patients with candidemia may be useful to assess the reproducibility of our results.

## Conclusions

An abdominal source of the infection is the most important factor significantly associated with the development of septic shock attributable to candidemia. In view of the prognostic implication of septic shock development, our results lead us to consider an appropriate stratification of candidemic patients on the basis of the source of the infection.

## Supplementary information

**Additional file 1.** Comparison between patients with and without intra-abdominal origin of candidemia.

## Data Availability

The datasets used and/or analyzed during the current study are available from the corresponding author on reasonable request.

## Abbreviations

CVC: Central venous catheter
ICU: Intensive care unit
OR: Odds ratio
