# Supplementary material for: Factors associated with the development of septic shock in patients with candidemia: a post hoc analysis from two prospective cohorts
Source: Crit Care. 2020 Mar 26;24:117. doi: 10.1186/s13054-020-2793-y (PMC7099832; doi:10.1186/s13054-020-2793-y)
Supplement: Supplementary file 1 — Additional file 1. Comparison between patients with and without intra-abdominal origin of candidemia. [file 13054_2020_2793_MOESM1_ESM.docx]

**Additional file 1.** Comparison between patients with and without intra-abdominal origin of candidemia

| **VARIABLE** | **Non IAI**  **N= 278 (%)** | **IAI**  **N=39 (%)** | **p-*value*** |
| --- | --- | --- | --- |
| **Demographics**  Sex, male  Mean age (± SD), years  Age ≥50 years  Median hospital stay until *Candida* BSI (IQR), days | 173 (62.2)  65.0 ± 17.4  238 (85.6)  16.5 (7 -31) | 24 (61.5)  67.9 ± 13.4  37 (94.9)  15 (7-31) | 1  0.33  0.13  0.96 |
| **Hospital ward stay at the time of candidemia onset**  General ward  Intensive care unit | 226 (81.2)  52 (18.7) | 28 (71.7)  11 (28.3) | 0.13  0.13 |
| **Charlson comorbidity index** | 3.3 ± 2.7 | 3.6 ± 2.8 | 0.55 |
| **Underlying condition**  Solid tumour  Cardiovascular disease  Diabetes mellitus  Chronic Lung disease  Chronic kidney failure  Chronic liver disease  Solid organ transplantation  Haematological malignancy  Haematopoietic stem cell transplantation | 93 (33.5)  91 (32.7)  82 (29.5)  47 (16.9)  41 (14.7)  35 (12.6)  23 (8.3)  18 (6.5)  9 (3.2) | 21 (53.8)  11 (28.5)  8 (20.5)  4 (10.3)  9 (23.1)  6 (15.4)  4 (10.3)  2 (5.1)  0 (0) | **0.02**  0.71  0.34  0.36  0.23  0.61  0.76  1  0.60 |
| **Risk factors for candidemia**  Previous antibiotic therapy  Central venous catheter  TPN during candidemia  Previous corticosteroid therapy  Abdominal surgery  Immunosuppressive therapy  Neutropenia | 259 (93.2)  202 (72.7)  138 (49.6)  65 (23.4)  59 (21.2)  39 (14.0)  9 (3.2) | 38 (97.4)  23 (59.0)  20 (51.3)  7 (17.9)  26 (66.7)  8 (20.5)  1 (2.6) | 0.49  0.09  0.54  0.87  **<0.001**  0.33  1 |
| **Previous antifungal treatment** | 43 (15.5) | 6 (15.4) | 1 |
| **Septic shock due to candidemia** | 79 (28.4) | 20 (51.3) | **0.006** |
| **Candida species**  *C. albicans*  *C. glabrata*  *C. parapsilosis*  *C. tropicalis*  *C. krusei*  *C. lusitaniae*  *C. auris*  Other | 114 (41.0)  47 (16.9)  50 (18.0)  25 (9)  6 (2.2)  4 (1.4)  27 (9.7)  11 (3.9) | 20 (51.3)  11 (28.2)  5 (12.5)  4 (10.3)  0 (0)  0 (0)  0 (0)  2 (5.1) | 0.23  0.12  0.50  0.76  1  1  0.06  0.64 |
| **Adequate initial antifungal treatment** | 106 (38.1) | 24 (61.5) | **0.008** |
| **Adequate source control of the infection*** | 109/251 (43.4) | 15/37 (40.5) | 0.86 |
| **Length of antifungal therapy, median, days (IQR)** | 17 (14-22) | 17 (9-22) | 0.73 |
| **30-day mortality** | 53 (19.1) | 17 (43.6) | **0.001** |

* the source of the infection was susceptible of control in 288 patients
